# Roentgen Rays—Their Application to Medicine and Surgery

**Published:** 1896-11

**Authors:** J. Clifford Perry

**Affiliations:** Passed Assistant Surgeon U. S. Marine Hospital Service.


ARTICLE III.

### ROENTGEN RAYS—THEIR APPLICATION TO MEDICINE AND SURGERY.

BY J. CLIFFORD PERRY, M. D.,

Passed Assistant Surgeon U. S. Marine Hospital Service.

Read before the Oregon State Medical Society, June 10, 1896.

I feel honored to have this opportunity of addressing you on a subject that has created such a widespread interest, and one that promises to be of so great a value to us as physicians and surgeons. When, as men's minds notwithstanding the important discoveries of the past two decades, were beginning to feel that too little was known of the unknown, Roentgen thrilled the scientific world by announcing his discovery; and as its possibilities unfold before the vivid imagination, an increased enthusiasm is awakened in the thousands of experimentalisc that are now working for the perfection of a discovery that promises so much.

No other fact in modern times has so shaken the general apathy of the public and of the press, that pertains to all strictly scientific subjects; and not even the results of Pasteur's virus and of Behring's serum have been read with the same avidity.

Before considering Roentgen's experiments, it may be advisable to briefly describe the results accomplished by some of the earlier investigators.

Crooke, in 1879, was the first to carry on systematically a series of interesting experiments on the behavior of electrical discharges through tubes of different vacua. In tubes of one-thousandth of the atmospheric pressure the electric discharge passes in a continuous stream from one terminal to the other, and a violet light is produced. The discharge is probably in the nature of electric conduction. The character of the discharge in a tube of high vacuum, one millionth of the pressure of the air, which are called Crooke's tubes, after the celebrated experimenter, changes markedly; it is no longer continuous between the cathode and anode, but disruptive in character.

Crooke applied the term of "radiant matter" to the rarefied air or vapor in his tubes, and assumed that in this high vacuum the molecules were free to move with immense velocity and through sensible distances without striking other molecules. He thought they were given off from the cathode, moved in lines normal to its surface, and were only stopped by collision with other molecules, a screen on the walls of the tube. He demonstrated that this matter moved in straight lines; could be defected with a magnet; and its bombardment on the walls of the tube produced heat.

Several years later Hertz, in conducting experiments with Crooke's tubes, ascertained that the effects were not confined to the tube, but were noticeable outside, and that these rays produced flourescence in certain substances. He supposed it was "radiant matter" projected through the glass.

In 1894 Professor Lenard showed that the effects observed outside the tube might take place some distance from it. He demonstrated that it was not "radiant matter," but was due to vibrations in the ether produced by the rays emanating from the cathode. He also ascertained that they would affect a photographic plate. Lenard thought all his rays were cathode; he was not aware of the new ray with new properties, and did not grasp the key that may possibly unlock the most obscure chambers of science.

Only a few months have elapsed since Professor Roentgen announced his discovery, and already much has been accomplished in showing the usefulness of the new photography.

The rays are formed at those portions of the tube that become most phosphorescent under the bombardment of the cathode rays, proceeding from these points in all directions. The new rays will penetrate nearly all substances, but in different degrees, depending upon the density of the material. Water and other liquids are very transparent, and hydrogen is markedly more so than air. Of the metals magnesium is the most permeable and lead the least so. Platinum intercepts practically all the rays; then comes next in order, gold, silver and copper, respectively. Aluminum offers little resistance to their passage, but glass, which is quite transparent to light, is very opaque to the new rays, only a small percentage of those formed passing out of the tube.

It is a question whether the retina is sensitive to these rays, since in the normal eye the crystaline lens is very opaque to them. But Dr. Brandles of the University of Halle, in some experiments with patients in whom both lenses had been removed for cataract, ascertained that they experienced a sensation of light. To demonstrate that it was the effect of the new rays and not those of ordinary light; he placed a sheet of aluminum between tbe source of the rays and their eyes and no change was noticeable, while a plate of glass arranged in the same manner prevented this sensation. Again the supjects were placed in a hood that intercepted all the light rays, and still they experienced the same sensation. He was not able to determine whether the effect was due to flourescence of the retina or to the action of the rays on the nerve endings. Professor Salvioni has produced flourescence in the retina of a cat's eye.

Roentgen and other experimenters have tried to refract these rays; investigations with prisms showed no deviation of them, while light rays were deflected from ten to twenty millimeters. They can not be refracted, thereby differing from those from the cathode.

Tesla and Professor Rood have succeeded in reflecting them, the former used zinc as a screen, the latter a sheet of platinum. Lenses prove ineffective in concentrating them.

There are many theories as to their nature. Professor Roentgen thinks they are longitudinal waves in the ether. Up to the present time we have supposed that ether only allowed the light waves to pass through, and we know that ordinary light passes by transverse waves. Others, among them Professor Wright, think they are transverse waves of very short length, moving with immense velocity and existing in the ultra-violet. Salvioni believes the rays are matter and not light. Mr. Edison considers them more in the light of static thrust than of undulatory propagations and thinks that their effect on the photographic film is due rather to direct mechanical than to chemical action.

The apparatus used in this line of work is expensive. A large induction coil, one giving a spark of 6-12 inches yields the best results. The tube is the most important factor in producing satisfactory results, and as there are many inferior ones on the market, none should be purchased that have not been thoroughly tested. The focus tube and the double reflector type, when properly excited, make excellent skiagraphs in a very short time, and are the most desirable.

No matter how excellent a tube works it becomes exhausted sooner or later. Some become temporarily fatigued and will again give good results if they are allowed to rest, or are subjected to a high temperature in an analling oven; this condition is caused by the destruction of some of the residual gas, and consequently, the production of too high a vacuum, which in all probability is due to the absorption of the gas by the electrodes, since those having only one terminal entering the tube have a longer life.

The tube is best excited by a high frequency alternating current and I use that of the incandescent light circuit, which is one of 100 volts, resistance being interposed to reduce the quantity of the current to the proper amount. If a Tesla coil is not used, which is better, a Tesla transformer and converter should be placed in the circuit. The advantages of this are: (1) An increase in the number of oscillations per second; (2) it prevents to a great extent the excessive heating of the tube and (3) it causes the production of Roentgen rays of greater intensity.

It is an advantage to have a phosphorescent screen, since it enables you to tell if your tube is producing the proper rays; and it is also excellent for diagnostic purposes, as it is only necessary to place the object between the tube and the screen, when the chemical coating becomes flourescent, and the picture is seen with the eye.

The phosphorescent screen is the outcome of the phosphorescent tubes. It having been known for some time that various substances placed in tubes and hermetically sealed, would, when exposed to the sunlight or that produced by burning magnesia, emit in the dark various colored rays. The light is called phosphorescence and the substances capable of developing it are known as phosphori.

The most rational theory suggested to explain these phenomena is that the undulations of the light convey their own vibratory motion to the phosphorescent materials and the light emitted from them is due to the vibration in their particles.

This is the general principle underlying the phosphorescent screen of the cryptoscope of Professor Salvioni and the flouroscope of Mr. Edison. The latter observer has examined 1800 substances, of which 72 flouresce, the best thing tungstate of calcium, which is six times as powerful as the platinum barium cyanide, and is the agent used in the construction of his apparatus.

After these preliminary remarks I will now consider the application of the new photography to medicine and surgery. It is an interesting subject and one that promises to be of special value to the surgeon. I will not attempt to relate the results of the various experiments, but will briefly give some of the most important achievements, and endeavor to point out some general lines of research in which the usefulness of these rays have been demonstrated. Although only a few months have elapsed, the apparatus has been so improved that the time of exposure has been considerably reduced, and the results obtained far more satisfactory; and with the perfection of the tubes and with more knowledge of the process, few parts of the human organism will withstand this invisible force.

The first application of Roentgen photography in surgery was in detecting foreign bodies embedded in the tissucs, the location of which could not be diagnosed.

Numerous experimenters have succeeded in skiagraphing these bodies, and with the picture for a guide the surgeon has found it easy to remove them. The presence of all metalic substances, as well as glass, in the tissues is quickly revealed by radiography. Mr. Sidney Rowland (British Medical Journal, March, 189[illegible],) reports an interesting case of a girl 6 years old, who had swallowed a coin, and as she suffered with colic, etc., it was supposed that the foreign body had lodged in the intestinal tract. A skiagraph was taken, which revealed that the coin had lodged at the ileo-coecal valve.

With the flouroscope the existence of metallic foreign bodies in the larynx can be quickly and satisfactorily diagnosed, thereby much facilitating their removal. Dr. J. Mount Bleyer has skiagraphed an intubation tube that had slipped into the trachea; with the picture for a guide, tracheotomy was performed and the tube removed.

I have taken skiagraphs showing the location of glass in the hand; and in another case in which the presence of glass was suspected I was able to demonstrate that no foreign body existed in the tissues, and that the painful nodule was due to cicatrical formation. I have also diagnosed a portion of a needle in the hand, its presence being quickly revealed by the aid of the new rays. Probably the most interesting case in which I have yet employed radiography for detecting bullets, is that of a young man who was accidentally shot in the neck several years ago. The skiagraph not only revealed the exact location of the bulletin, but also showed the cervical vertebrae quite distinctly.

This process is also an important aid in studying the injuries and diseases of the osseous system. It is often difficult to determine between a fracture and a dislocation, but Roentgen rays come to your assistance and the problem is easily solved. Mr. Edward Gray ("British Medical Journal," March 7, 1896,) reports a case in which the injury to the elbow joint was quite obscure, and as there was free flexion and extension, and it was impossible to make a satisfactory diagnosis by ordinary means, a skiagraph was taken, which revealed a rare dislocation, both bones of the forearm being displaced outward.

In this connection, I will briefly describe the following case: A boy 14 years old received an injury to the elbow point; there was some doubt about the diagnosis, but his physician treated him for fracture. One month later the patient passed under the care of another physician and the case was referred to me to make a skiagraph of the injured member. The picture showed an intercandyloid fracture and also a partial dislocation of the bones of the forearm backward.

After reducing fractures and applying the proper dressings, if there is any doubt about the correct opposition of the fragments, a skiagraph can be taken without disturbing the dressings, as they do not impede the passage of the rays, and it can be determined whether it is necessary to readjust the bone. The line of fracture can be mapped out in old cases, even after months have elapsed. Skiagraphs of the deformity resulting from improper treatment have already been brought forward as evidence in damage suits and no doubt they will be much used in medico-legal cases.

Another use for this photography is in diseased conditions of the bones. Several operators have obtained excellent results in locating the affected area in tubercular disease of the joints; the diseased portion is more permeable to the rays and, consequently, the shadow it produces is less dense than that formed by the surrounding healthy bone; even a small area of unhealthy osseous tissue is generally clearly outlined, and the surgeon is informed of its extent and location before attempting an operation. The cartilagenous portions of the joints cast no shadow, as they are transparent to the rays.

Dr. Keen ("American Journal Medical Sciences," March, 1896,) reported a case of tubercular disease of the elbow joint in which the diseased portion of both the humerus and the ulna was clearly shown. Mr. Parker ("British Medical Journal," May 10, 1896,) reports an interesting case of a boy apparently suffering with tubercular disease of the elbow joint, in whom a skiagraph revealed the presence of a carious area the size of an almond in the upper end of the ulna. This defect in the bone had not been suspected, and it was verified by an operation.

Diseases of the ankle, knee and other joints have been successfully photographed. Abscesses in the bone can also be diagnosed by this new aid.

The presence of exostoses and other tumors of the bone are easily demonstrated by the use of these rays. They are also of marked advantage in detecting separation of the epiphyses and in diagnosing green stick fractures.

Skiagraphs of the entire skeleton have been taken, showing plainly the vertebrae and the intervertebral substance, also showing faintly the heart and liver.

It is natural to think of using this means for detecting hepatic, renal and vescical calculi. It has been demonstrated that gall stones are easily penetrated by these rays, and, consequently, it is very problematical that they could be photographed in the gall bladder. On the other hand, renal and vescical calculi are very opaque to them, and Professor Neusser of Vienna has successfully photographed renal stones in the living subject.

The application of this photography as a diagnostic aid to the obstetrician has been carefully considered in a paper by Dr. Davis ("American Journal Medical Sciences," March, 1896.) He has succeeded in obtaining skiagraphs of the fœtus in utero, in which its position was fairly well outlined. He thinks it is possible to ascertain whether more than one fœtus is present and that contracted pelves and abnormalities in the fœtus can be determined by its use.

So far Roentgen photography has not proved of special value in strictly medical subjects; still Dr. F. H. Williams in a paper read before the last meeting of the American Medical Association, stated that with the flouroscope he was able to map out the consolidated areas of lung tissue in pneumonia and tubercle, those portions showing darker on the screen than the surrounding healthy structure.

Dr. Levy by means of a tube devised by himself, which produces rays of great penetrating power, has been able to diagnose by means of the flouroscope hypertrophy of the heart, and to distinctly see the liver and lungs.

The tissues are of different density, and if the exposure is not too long the different layers can be detected in the picture. I have made skiagraphs showing distinctly the skin, fat, fascia, muscles, bones, and the medullary canal in the bones.

Numerous experimenters have investigated the action of the rays on bacteria, but so far they have not had any deleterious effect upon the microbes. Professor Delepine has reported some experiments he and Professor Schuster made concerning their germicidal action. Tubes containing growths of cholera vibrio, bacillus coli communis, typhoid fever and anthrax were exposed to the rays for two hours—control tubes were kept under the same conditions (excepting exposure to the rays). From each of these tubes, other tubes containing sterilized culture medium were inoculated and no difference could be detected in the growth of the organisms which had been exposed to the rays and those that had not. Dr. Kyle of Philadelphia, and many others, have also investigated this subject and have obtained nothing but negative results.

From the reported results of others and from my individual experiments, I am led to conclude as follows:

First—That Roentgen rays are of inestimable value in detecting and locating foreign bodies in the tissues.

Second—That they are important as a diagnostic aid in diseases and injuries of the osseous system.

Third—That they have no germicidal action.—*M. S.*

